# Hands-Free Conventional Radiographic Ventrodorsal Hip Extended View

**DOI:** 10.3389/fvets.2020.00286

**Published:** 2020-06-10

**Authors:** Ana Santana, Sofia Alves-Pimenta, João Martins, Bruno Colaço, Mário Ginja

**Affiliations:** ^1^Faculty of Veterinary Medicine, Lusófona University, Lisbon, Portugal; ^2^CITAB - Centre for the Research and Technology of Agro-Environmental and Biological Sciences, University of Trás-os-Montes and Alto Douro, Vila Real, Portugal; ^3^Department of Animal Science, University of Trás-os-Montes and Alto Douro, Vila Real, Portugal; ^4^Department of Veterinary Science, University of Trás-os-Montes and Alto Douro, Vila Real, Portugal

**Keywords:** canine hip dysplasia, Norberg angle, reproducibility, hind limb holder, ventrodorsal hip extended view

## Abstract

Hip dysplasia (HD) is an important hereditary orthopedic disease in the dog associated with osteoarthritis and inadequate welfare for affected animals. The radiographic ventrodorsal hip extended (VDHE) view is used worldwide to select the better animals for breeding. This view normally is performed with manual restraining of the dog to obtain radiographs with acceptable technical quality. The veterinarian exposition to ionizing radiation is inevitable. In this study, the technical quality of VDHE radiographs and hip measurements was compared in 65 dogs radiographed twice, one with the common veterinarian manual restraining and the other obtained using a hind limb holder device, without the veterinarian within the X-ray room. The variables studied were pelvic tilting, patella displacement index, Norberg angle (NA), and subluxation hip category. The results showed a random distribution of right and left pelvic tilting, patella lateral or medial displacement, and hip subluxation categories in both samples (*P* > 0.05). The holder device positioning showed a better pelvic symmetry (*P* < 0.05) and a similar patellar displacement (*P* > 0.05). The mean ± standard deviation of NA was 101.1° ± 6.2° and 100.9° ± 6.1° in the manual and holder device hind limb restraining, respectively (*P* > 0.05), and the lower limit of 95% confidence interval of intraclass correlation coefficient was >0.75. These results showed statistical reproducibility of NA measurements by the hind limb holder device, and the examiner was protected from exposure to ionizing radiation within the X-ray room.

## Introduction

Hip dysplasia (HD) is an important hereditary orthopedic disease in the dog associated with osteoarthritis, resulting in an inadequate welfare for affected animals (1, 2). The recommended medical strategy to reduce HD's negative impact on canine populations is to select the better animals for breeding (3, 4). Despite its determinant hereditary component, a genetic test that permits a reliable diagnosis does not yet exist, and it is based on the radiographic examination (3–6). Canine HD phenotype inherence is considered highly complex (7). The radiographic pelvic view must comply with positioning rules to obtain the adequate quality for medical radiographic analysis (1). The conventional ventrodorsal hip extended (VDHE) view is the most used worldwide (2–4, 8, 9). In this view, the dog is sedated or anesthetized and placed in dorsal recumbency on the X-ray table, and the examiner maintains the dog's hind limbs extended parallel to each other and the stifles internally rotated (8). The medical objective is to obtain a radiograph with symmetrical pelvis and parallel femurs and patella centered on the distal femoral metaphysis (8, 9). Thousands of these types of radiographs are taken daily. The permanence of the examiner within the X-ray room to hold the animal leads inevitably to his exposition to ionizing radiation (8). The interaction of primary X-ray beam with animal origin scatter radiation disperses in random directions in the X-ray room (10). Currently, in England, animal physical restraint in the X-ray room is not allowed unless there is a clinical reason that contraindicates restraint by any other means (11). Thus, the British Veterinary Association has specific recommendations of dog positioning for radiographic hip evaluation (11–13). Precautions must be taken to reduce the possible harmful effects of ionizing radiation to the examiner (10). The ALARA principle “as low as reasonably achievable” for ionizing radiation exposure is a concept in the national and international radiation safety regulations (10).

The main aim of this work was to compare the technical quality of VDHE views obtained using a hind limb holder device fixer with similar views in same animals obtained with the conventional examiner physical restraining. For this purpose, the pelvis symmetry, degree of femoral rotation, Norberg angle (NA), and hip subluxation category (SC) were evaluated. As far as the authors know, there is no published work that has made this comparison; nor is there any similar holder device for the hind limbs to be used for this purpose.

## Materials and Methods

In this prospective study, 65 dogs (36 females and 29 males) were used from five different Portuguese breeds (28 Portuguese pointer dogs, 27 Estrela mountain dogs, 5 Transmontano cattle dogs, 4 Rafeiro do Alentejo, and 1 Barbado da Terceira). These dogs were presented at the Veterinary Teaching Hospitals of University os Trás-os-Montes and Alto Douro (UTAD) or University Lusófona de Humanidades e Tecnologias in the years of 2018 and 2019 for screening HD. Recorded data included breed, age at time of the radiography, sex, and body weight. The inclusion criteria were dogs older than 4 months, with normal musculoskeletal development in clinical examination, with pairs of VDHE views: one with manual restraining and the other with the hind limbs holder device. Radiographs should have an adequate technical quality for canine HD scoring, with maximum pelvic tilting of 3 degrees and a patellar displacement index from the femoral metaphysis center <0.15 (lateral or medial) (14). The minimum sample size was estimated using a *t*-test table, selecting a statistical significance of 0.05, a medium variable effect size (0.5), and a statistical power of 0.8, and resulted in a sample of 64 observations (15).

All examinations were performed with the dog owner's consent, and all the animal procedures undertaken as part of the work described in this work were performed in compliance with the regulations of our institutions (n° 1044-e-DCV66 2018) and in accordance with the Portuguese and European regulations for animal use and care (European Directive 2010/63/EU and National Decree-Law 113/2013).

### Radiographic Procedures

**Figure 1:**
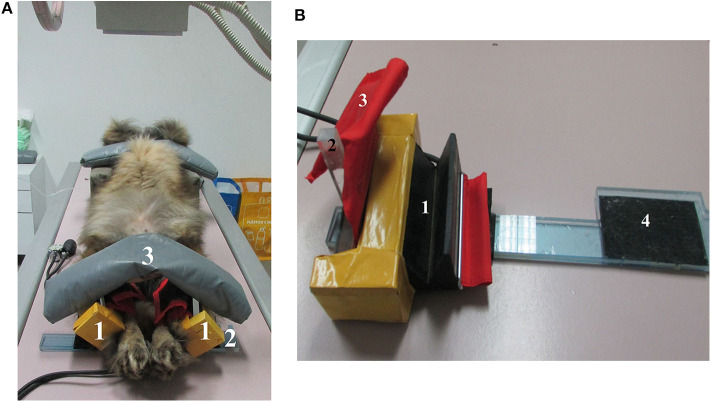
**(A)** Estrela mountain dog, female, sedated on an X-ray table to obtain the hands-free ventrodorsal hip extended view. (1) Holder device to fix both hind limbs in dog tarsus, (2) acrylic base coated with self-adhesive Velcro, (3) cylindrical sandbag. **(B)** Holder device to fix the dog's tarsus. (1) Rubber groove to accommodate the dog tarsus, (2) acrylic stem that will be attached to the contralateral to maintain hind limb medial rotation, (3) sphygmomanometer to fix the tarsus firmly in rubber groove, (4) acrylic base coated with self-adhesive Velcro to maintain the holder device in position.

The radiographs were performed, with dogs under deep sedation using medetomidine (Domitor; Orion Corporation, Espoo, Finland) and butorphanol (Torbugesic Injectable; Fort Dodge Veterinaria, Girona, Spain) intravenously. The sedation was reversed with atipamezole hydrochloride (Antisedan; Orion Corporation) intramuscularly. In each animal, two VDHE views in the same sequence were obtained: first the VDHE view with dogs in dorsal recumbency on the X-ray table and the examiner positioning hind limbs in extension and rotated medially (8); and in the second, VDHE view was hands-free where the dog was placed on the X-ray table in a similar position and the rear limbs placed in extension and rotated medially using a holder device (Figure 1). This holder device has a rubber groove to fit the dog's tarsus, which was subsequently fixed firmly in each rear limb using a sphygmomanometer with air at 120 mm Hg. Another important component of the positioner is an acrylic stem that is then fixed to the contralateral (we used adhesive strip) to eliminate the supination natural hind limb's force. To complete the limbs fixation under the holder devices, an acrylic base was used coated with a self-adhesive Velcro and on top of everything a cylindrical sandbag of 4 kg (Figure 1). The sequence of procedures of this view was as follows: (1) a holder device was fixed firmly on each tarsus of the dog; (2) the examiner put the rear limbs of the dog as if it were to be performed common VDHE view; (3) an assistant placed the acrylic bases under the holder devices; (4) the assistant attached the acrylic stem of the right and left holder devices and placed on top the cylindrical sandbag to maintain rear limbs on medial rotation and extension; (5) the examiner and the assistant left the dog on the X-ray table and went away from the X-ray room.

### Radiographic Measurements

The radiographs were obtained in DICOM format using the computed digital radiography Fujifilm FCR Prima reader unit. The pelvic symmetry was evaluated measuring in millimeters the right and left iliac horizontal diameter (IHD) drawing a straight line between the dorsal and ventral iliac cortex at the level of the cranial aspect of sacroiliac joint on right and left sides (16). The IHD asymmetry in millimeters (*x*) was used to estimate the degrees of pelvic tilting (*y*) using the regression equation *y* = 0.997*x* + 0.06 (16).

The horizontal distance between the patellar central vertical axis line to the femoral lateral and medial cortex is used to evaluate if it is centered (same distance to the lateral and medial femoral cortex), external rotated (closer the lateral cortex), or internal rotated (closer the medial cortex) (17). The lateral or medial patellar displacement distance was measured in millimeters from the femoral center, and the respective displacement indices were calculated dividing these distances by the metaphysis thickness (14, 17).

The NA was measured in degrees as the angle formed by one line drawn between the centers of the femoral heads and the other from the center of the femoral head to the craniolateral aspect of the acetabular rim (18). The SC was classified from 0 to 6 femoral head evaluating joint congruence and the relationship between the position of femoral head center and the dorsal acetabular edge (13, 14).

The radiographic measurements were performed on randomly chosen digital images of each set; the positioning variables (pelvic tinting and patella displacement index) were measured by J.M., and the HD parameters (NA, SC) by M.M.G., using the software OSIRIS (OSIRIS Imaging Software version 3.1: University Hospital of Geneva, Geneva, Switzerland).

### Statistical Analysis

Statistical analysis was performed using the SPSS (SPSS Statistics for Windows version 23.0; IBM Corp., Armonk, NY, USA). Descriptive statistics were performed for all continuous variables. The data analysis was performed at individual joint level.

The χ^2^ test of independence was used to determine if there was a significant relationship between the slight right or left pelvic tilting of each set of radiographs, considering pelvis symmetry when the tilting was <1 degree. This test was also used to evaluate the distribution of the slight lateral or medial patella displacement in each set, considering the patella centered when its displacement from the center was <1 mm. The χ^2^ test was still used to evaluate the distribution of SCs in each set. The null hypothesis was that there was no relationship between the methodology used in each set of radiographs and the variables distribution (19).

The intraclass correlation coefficient (ICC) and the Bland–Altman analysis were used to study the repeatability of the NA, pelvic tilting, and patellar displacement on examiner and holder device dog's positioning (20). An ICC of 1 indicates perfect agreement, and an ICC of 0 indicates no agreement. A lower limit 95% confidence interval (CI) of ICC >0.75 was defined as an adequate correlation (20). To determine the limits of agreement (LA) according to the Bland–Altman method, we calculated the mean difference ($\bar{d}$) between pairs of measurements and its 95% CI as $\bar{d}$ ± 2 standard error of the mean. When this interval includes zero, measurements are considered to be in agreement (19, 21). Then, 95% LAs were estimated as $\bar{d}$ ± 1.96 standard deviation. Narrower 95% LA is associated with higher agreement between methods. The statistical power was estimated to evaluate the ability of our research design to detect variable differences between groups (15). The *P* < 0.05 was considered to be significant (19, 21).

## Results

**Figure 2:**
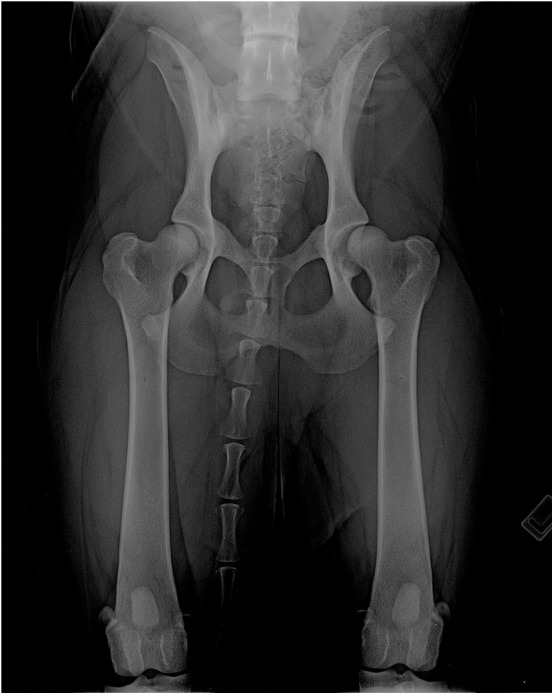
Hands-free ventrodorsal hip extended radiograph of an Estrela mountain dog, female.

Sixty-five pairs of VDHE views (130 hip joints) were available from manual-retrained and hands-free holder device view sets (Figure 2). The age of dogs ranged from 4 to 93 months [mean ± standard deviation (SD), 24.4 ± 20.2 months], and the body weight ranged from 14 to 68 kg (mean ± SD, 29.9 ± 12.8 kg). The χ^2^ test of independence null hypothesis was accepted for pelvic tilting, patellar medial and lateral displacement, and SCs in the comparison between both sets of images (Table 1).

**Table 1:** Pelvic tilting, patella displacement, and subluxation categories in manual-restraining and hands-free holder device.

	**Manual restraining**	**Free-hand**	**χ^**2**^ test**
		**holder device**	
Pelvis			*P* = 0.98
Symmetry^*^	34	35	
Tilting to the right	12	12	
Tilting to the left	19	18	
Patella^**^			*P* = 0.44
Centered	35	36	
Lateral displacement	52	60	
Medial displacement	43	34	
Subluxation category^***^			*P* = 0.80
1	17	21	
2	68	65	
3	33	35	
4	12	9	

* Pelvis symmetry was considered for tilting <1 degree.

** Patella centered was considered for lateral or medial patella displacement <1 mm.

*** *The subluxation categories of 0, 5, and 6 were not used*.

**Figure 3:**
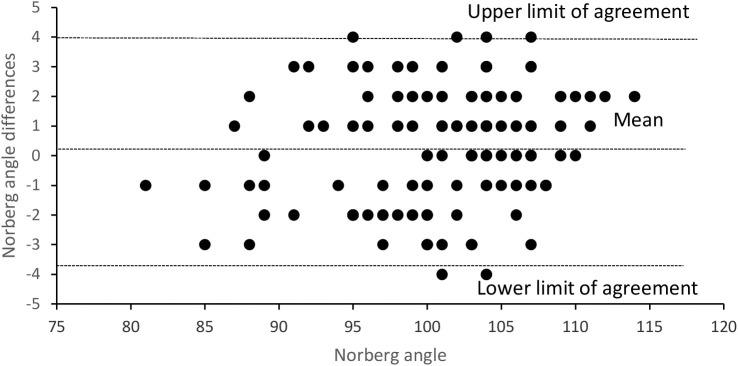
Differences between Norberg angle on examiner restraining radiographic views and on hands-free holder device views plotted against the Norberg angle examiner results.

In the manual-restraining views, the mean ± SD pelvic tilting, patella displacement index from the center, and NA were 1.4 ± 1.0°, 0.05 ± 0.04, and 101.1 ± 6.2°, and in hands-free holder device views were 0.9 ± 0.9°, 0.05 ± 0.04, and 100.9 ± 6.1°, respectively. The variable paired differences and the statistical power results are described in Table 2 and Figure 3. The ICC for single measures was significant in all cases (*P* < 0.05) with the following values: 0.47 (95% CI, 0.26–0.64), 0.42 (95% CI, 0.27–0.55), and 0.95 (95% CI, 0.92–0.96) for pelvic tilting, patella displacement index, and NA, respectively.

**Table 2:** Paired variable differences between examiner and hands-free holder device dog's positioning.

**Variable**	***n***	**Paired differences**					***P***	**Effect size**	**Power**
		**Mean**	**SD**	**SEM**	**95% CI**				
					**Lower**	**Upper**			
Pelvic tilting (°)	65	0.38	0.95	0.12	0.14	0.61	<0.05	0.39	0.88
PDI	130	0.03	0.05	0.00	−0.01	0.01	>0.05	0.05	0.09
Norberg angle (°)	130	0.23	1.96	0.17	−0.11	0.57	>0.05	0.12	0.27

*CI, confidence interval; n, number; P, statistical significance; PDI, patellar index displacement; SEM, standard error of the mean; SD, standard deviation*.

## Discussion

Radiography has been used in the diagnosis of HD in dogs worldwide for more than 50 years; there are databases with more than 1 million animals (2, 3, 5, 13). Currently, the main veterinary strategy to reduce the impact on HD in canine populations continues to be based on radiographic diagnosis and breeding selection (3, 4). The main radiographic view used worldwide is the VDHE view, with thousands of these radiographs being taken daily, and in all of them, the dog is positioned by the veterinarian, except in the United Kingdom (2, 3, 13). Here animal physical restraint in the X-ray room is not allowed for HD diagnosis, and some hands-free methodologies based on the use of ropes are available (12, 13). We are advocates of this radiographic approach because we think that whenever possible the ALARA principle in veterinary medicine should be respected. The effect of even low levels of ionizing radiation may accumulate and could represent a potential health hazard (10). However, no study has been able to assess the role of specific low-ionizing radiation exposures in cancer risk (22). A limitation of this study is that the authors do not have practical experience with other hands-free radiographic methodologies; we think that it is important to disseminate these alternative procedures, and we hope in the future to get some free-will followers.

Previous works have shown different levels of longitudinal lateral pelvic tilting and femoral internal or external rotation association to inadequate NA, femoral head subluxation index, or femoral head SC measurements (9, 14, 17, 23). Other showed that longitudinal craniocaudal pelvic tilting does not affect measurement of NA (24). However, the recommended positioning without rotations and according to the standard should be always a fundamental objective in radiographic technique (1, 9), although perfect radiographs are scarce and some level of body rotation is acceptable for HD scoring purposes (9, 14). Normal hips must have good femoral head and acetabular congruence with NA ≥105° and low SC (1, 13). However, other studies argue that the NA cutoff for normal hips should be larger at ~110°, to maximize the specificity of the diagnosis of non-dysplastic hips (25).

The observed slight pelvic tilting and patellar displacement index in our sample is normal and similar to other studies (14). The smaller pelvic tilting (*P* < 0.05) in hands-free holder device views is a positive and desirable effect. The non-equivalent force applied by the examiner on the dogs' left and right hind limbs can be associated to some slight VDHE view asymmetries (26). The similar patellar displacement index in a random distribution not associated with the used method to obtain radiographs is important, as it indicates the good functionality of the use of hands-free VDHE view methodology and having no bias (19). However, the ICC for this variable is low and indicates that there is no true reproducibility (20), but we think that this is perfectly normal and would also happen if this variable was studied in terms of intraobserver variability. One aspect that should be valued is the obtained statistical reproducibility of NA, *P* > 0.05 on paired differences and ICC 95% CI lower limit of 0.92, and the equivalent distribution of the SC, variables determinants for the HD grading in the scoring schemes of the Fédération Cynologique Internationale's and the British Veterinary Association/Kennel Club (1, 13, 20). However, the low statistical power of the sample does not allow concluding that there is no significant difference between groups (15). As the size effect is very small (0.12), we will need a sample with approximately 1,000 animals to obtain enough statistical power (0.80) to demonstrate that NA differences are not associated to the used methodologies (15). In medical studies, when the investigated differences are very small, they can be considered with no clinical importance, and it is not worth to detect their origin (27). In the extreme, when the mean of the differences is 0, there is no statistical power that can be used, because the required sample is infinite.

The heterogeneity of the sample associated to the use of medium and large breeds of dogs, different ages, and different examiners should be seen as a positive aspect of the study and methodology, because it allows highlighting all these possible potentialities of the hind limb holder device. However, the small number of breeds and their low representativeness in global terms can be also mentioned as an additional limitation of this study. The hands-free procedure described here is not traumatic for the animal, in contact with its tissues, there is only rubber and the sphygmomanometer, which are both non-traumatic soft materials. The hind limb holder device also does not exert any additional force; it is simply intended to provide adequate stability of the limbs, which are previously positioned by the examiner. In terms of procedure, two people are essential: the examiner to place adequately the dog in VDHE view and an assistant to properly stabilize the holder device. In future studies, it might be interesting to test this holder device and associated technique with different operators, other dog breeds, with and without experience to evaluate the ease of the procedure and the interobserver repeatability, as well as to perform a comparison with the restraint method used in the United Kingdom, the international reference of the hands-free VDHE view.

## Conclusions

This hind limb holder device and associated methodology showed reliability in dog's positioning on the X-ray table to perform the VDHE view, used to evaluate the HD grade. The procedure does not cause any harm to the animal. The holder device allowed obtaining radiographs with better pelvic symmetry and similar patellar displacement. The NA measurements showed statistical reproducibility in comparison with measurements on a manual-restraining set; however, the study design did not allow obtaining enough statistical power, because of the very small effect size. The use of the holder device allows protecting the examiner from exposure to ionizing radiation within the X-ray room.

## Data Availability Statement

The datasets generated for this study are available on request to the corresponding author.

## Ethics Statement

The animal study was reviewed and approved by Comissão de Ética—UTAD. Written informed consent was obtained from the owners for the participation of their animals in this study.

## Author Contributions

AS: acquisition of data and drafting of the manuscript. SA-P: acquisition of data and critical revision of manuscript. JM: acquisition of data, critical revision of manuscript, and radiographic measurements. BC: contribution to concept/design and critical revision of manuscript. MG: contribution to concept/design, acquisition of data, data analysis/interpretation, radiographic measurements, and drafting of the manuscript.

## Conflict of Interest

The authors declare that the research was conducted in the absence of any commercial or financial relationships that could be construed as a potential conflict of interest.

## References

[1] Ginja MM Silvestre AM Gonzalo-Orden JM Ferreira AJ. Diagnosis, genetic control and preventive management of canine hip dysplasia: a review. Vet J. (2010) 184:269–76. 10.1016/j.tvjl.2009.04.009 19428274

[2] Reagan JK. Canine hip dysplasia screening within the United States: Pennsylvania hip improvement program and orthopedic foundation for animals hip/elbow database. Vet Clin North Am Small Anim Pract. (2017) 47:795–805. 10.1016/j.cvsm.2017.02.003 28434528

[3] Ohlerth S Geiser B Flückiger M Geissbühler U. Prevalence of canine hip dysplasia in Switzerland between 1995 and 2016-a retrospective study in 5 common large breeds. Front Vet Sci. (2019) 6:378. 10.3389/fvets.2019.00378 31709271 PMC6821640

[4] James HK McDonnell F Lewis TW. Effectiveness of canine hip dysplasia and elbow dysplasia improvement programs in six UK pedigree breeds. Front Vet Sci. (2020) 6:490. 10.3389/fvets.2019.00490 32010712 PMC6974481

[5] Hedhammar A Olsson SE Andersson SA Persson L Pettersson L Olausson A. 318 canine hip dysplasia: study of heritability in 401 litters of German shepherd dogs. J Am Vet Med Assoc. (1979) 174:1012–6. 570968

[6] Ginja M Gaspar AR Ginja C. Emerging insights into the genetic basis of canine hip dysplasia. Vet Med. (2015) 6:193–202. 10.2147/VMRR.S63536 30101106 PMC6070022

[7] Mikkola LI Holopainen S Lappalainen AK Pessa-Morikawa T Augustine TJP Arumilli M. Novel protective and risk loci in hip dysplasia in German shepherds. PLoS Genet. (2019) 15:e1008197. 10.1371/journal.pgen.1008197 31323019 PMC6668854

[8] Ginja MM Silvestre AM Colaço J Gonzalo-Orden JM Melo-Pinto P Orden MA. Hip dysplasia in Estrela mountain dogs – prevalence and genetic trends 1991–2005. Vet J. (2009) 182:275–82. 10.1016/j.tvjl.2008.06.014 18722145

[9] Genevois JP Cachon T Fau D Carozzo C Viguier E Collard F. Canine hip dysplasia radiographic screening. Prevalence of rotation of the pelvis along its length axis in 7,012 conventional hip extended radiographs. Vet Comp Orthop Traumatol. (2007) 20:296–8. 10.1160/VCOT-07-01-0007 18038007

[10] Dowsett D Kenny PA Johnston RE The Physics of Diagnostic Imaging. London: CRC Press (2006).

[11] BVA Guidance Notes for the Safe Use of Ionising Radiations in Veterinary Practice. London: British Veterinary Association (2002).

[12] BVA Instructional Videos on Radiography Positioning. (2020). Available online at: http://chs.bva.co.uk/.

[13] Dennis R Interpretation and use of BVA/KC hip scores in dogs. In Pract. (2012) 34:178–94. 10.1136/inp.e2270

[14] Martins J Colaço B Alves-Pimenta S Gonzalo Orden JM Ferreira AJ Ginja MM. Effect of the dog positioning on X-ray table on hip dysplasia parameter evaluation. Vet Comp Orthop Traumatol. (2019) 32:376–82. 10.1055/s-0039-1688991 31127598

[15] Cohen J Statistical Power Analysis for the Behavioral Sciences. 2nd ed London: Lawrence Erlbaum Associates, Inc (1988).

[16] Martins J Colaço BJ Ferreira AJ Ginja MM. Analysis of pelvic rotation on the standard hip ventrodorsal extended radiographic view. Vet Comp Orthop Traumatol. (2016) 29:68–74. 10.3415/VCOT-15-02-0025 26548580

[17] Martins J Colaço BJ Alves-Pimenta S Gonzalo-Orden JM Ferreira AJ Ginja MM Femoral rotation and relationship between the femoral head and the acetabulum. Vet Med. (2017) 62:589–59. 10.17221/41/2017-VETMED

[18] Henricson B Norberg I Olsson SE. On the etiology and pathogenesis of hip dysplasia: a comparative review. J Small Anim Pract. (1966) 7:673–88. 10.1111/j.1748-5827.1966.tb04393.x 5342030

[19] Petrie A Watson P Statistics for Veterinary and Animal Science. Oxford: Blackwell Science (1999).

[20] Lee J Koh D Ong CN. Statistical evaluations of agreement between two methods for measuring a quantitative variable. Comput Biol Med. (1989) 19:61–70. 10.1016/0010-4825(89)90036-X 2917462

[21] Bland JM Altman DG. Statistical methods for assessing agreement between two methods of clinical measurement. Lancet. (1986) 1:307–10. 2868172

[22] Fritschi L. Cancer in veterinarians. Occup Environ Med. (2000) 57:289–97. 10.1136/oem.57.5.289 10769295 PMC1739954

[23] Martins J Colaço BJ Alves-Pimenta S Ferreira AJ Ginja MM Effects of pelvis rotation on projected radiographic position of femoral head in relationship to acetabulum. Vet Med. (2017) 62:377–85. 10.17221/127/2016-VETMED

[24] Bausman JA Wendelburg KL. Evaluation of the effect of pelvic tilt in the coronal plane on the Norberg angle measured in ventrodorsal radiographic views of a canine hip joint bone model. Am J Vet Res. (2010) 71:1348–53. 10.2460/ajvr.71.11.1348 21034326

[25] Gaspar AR Hayes G Ginja C Ginja MM Todhunter RJ. The Norberg angle is not an accurate predictor of canine hip conformation based on the distraction index and the dorsolateral subluxation score. Prev Vet Med. (2016) 135:47–52. 10.1016/j.prevetmed.2016.10.020 27931928

[26] Martins J Ferreira AJ Ginja MM. Morphometric assessment of the hip joint in the Estrela mountain dog breed. Vet Comp Orthop Traumatol. (2012) 25:202–10. 10.3415/VCOT-11-07-0101 22367104

[27] Jones SR Carley S Harrison M. An introduction to power and sample size estimation. Emerg Med J. (2003) 20:453–8. 10.1136/emj.20.5.453 12954688 PMC1726174

